# Identification and Characterization of Cell Wall Proteins of a Toxic Dinoflagellate *Alexandrium catenella* Using 2-D DIGE and MALDI TOF-TOF Mass Spectrometry

**DOI:** 10.1155/2011/984080

**Published:** 2011-09-05

**Authors:** Da-Zhi Wang, Hong-Po Dong, Cheng Li, Zhang-Xian Xie, Lin Lin, Hua-Sheng Hong

**Affiliations:** State Key Laboratory of Marine Environmental Science, Environmental Science Research Centre, Xiamen University, Xiamen 361005, China

## Abstract

The cell wall is an important subcellular component of dinoflagellate cells with regard to various aspects of cell surface-associated ecophysiology, but the full range of cell wall proteins (CWPs) and their functions remain to be elucidated. This study identified and characterized CWPs of a toxic dinoflagellate, *Alexandrium catenella*, using a combination of 2D fluorescence difference gel electrophoresis (DIGE) and MALDI TOF-TOF mass spectrometry approaches. Using sequential extraction and temperature shock methods, sequentially extracted CWPs and protoplast proteins, respectively, were separated from *A. catenella*. From the comparison between sequentially extracted CWPs labeled with Cy3 and protoplast proteins labeled with Cy5, 120 CWPs were confidently identified in the 2D DIGE gel. These proteins gave positive identification of protein orthologues in the protein database using *de novo* sequence analysis and homology-based search. The majority of the prominent CWPs identified were hypothetical or putative proteins with unknown function or no annotation, while cell wall modification enzymes, cell wall structural proteins, transporter/binding proteins, and signaling and defense proteins were tentatively identified in agreement with the expected role of the extracellular matrix in cell physiology. This work represents the first attempt to investigate dinoflagellate CWPs and provides a potential tool for future comprehensive characterization of dinoflagellate CWPs and elucidation of their physiological functions.

## 1. Introduction

The dinoflagellates are a diverse group of unicellular algae that comprise a large part of the marine phytoplankton [1]. They are not only important primary producers and an important part of the food chain in the marine ecosystem, but also the major causative species of harmful algal blooms (HABs) in the coastal zone [2]. Moreover, many dinoflagellate species can produce various potent toxins that impact human health through the consumption of contaminated shellfish, coral reef fish, and finfish or through water or aerosol exposure [3]. In the past few decades, much effort has been devoted to the study of HABs and dinoflagellate toxins. However, many aspects of them are still not well elucidated due to the unusual physiological and molecular features of dinoflagellates, and this has impeded our understanding of dinoflagellate-caused HABs and subsequently their monitoring, mitigation, and prevention [4]. 

**Figure 1:**
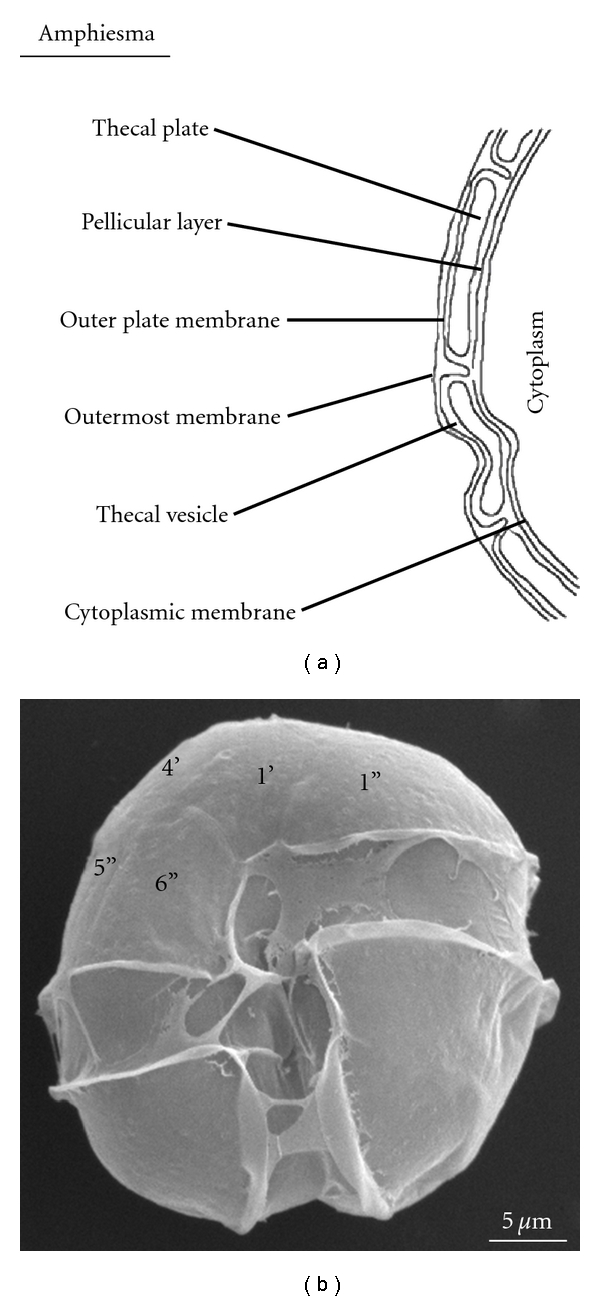
Schematic diagram of the amphiesma of a typical thecate dinoflagellate based on Morrill and Loeblich (1984). (a) Structure of the amphiesma, including a continuous outermost membrane, an outer plate membrane, a single-membrane bounded thecal vesicle, and a cytoplasmic membrane. Inside this vesicle, a number of cellulosic thecal plates are subtended by a pellicular layer. (b) Scanning electron micrograph of *A. catenella*, with the continuous outermost membrane obvious on the cell surface.

Dinoflagellates typically have an outer covering called the theca or amphiesma (Figure 1), which consists of a continuous outermost membrane, an outer plate membrane, and a single-membrane bounded thecal vesicle [5, 6]. Inside this vesicle, a number of cellulosic thecal plates are subtended by a pellicular layer. Thecal plates usually consist primarily of cellulose and polysaccharides with a small amount of proteins. Although much effort has been devoted to understanding the cell wall ultrastructure of dinoflagellates using electron microscopic and cytochemical approaches, molecular information on cell wall biogenesis and dynamics is lacking. 

It is known that a number of proteins and enzymes reside on the cell wall and outer membrane of phytoplankton, such as high-affinity binding proteins [7, 8], transporters [9–14], stress proteins [15], signaling proteins [16], and ectoenzymes [17–25]. These proteins play important roles ranging from nutrient utilization, defense, signaling, and cell adhesion to cell-cell recognition. The cell wall of dinoflagellates is a subcellular component of substantial interest with regard to various aspects of cell surface associated ecophysiology. However, there are few experimental data available for the cell wall of dinoflagellates compared with other organisms due to the lack of the whole genome. So far, only a limited number of cell wall proteins (CWPs) and enzymes have been identified and characterized at the biochemical and functional level, and neither the mechanism of their functions nor their locations have been elucidated [26–30]. A few studies indicate that cell wall-associated proteins and their activities are known to be induced or increased by factors limiting the growth of these members of the eukaryotic phytoplankton, because they may enhance cell scavenging of nutrients. Moreover, dinoflagellate CWPs may also be involved in signaling pathways [16]. Clearly, the cell wall presents an important site of interaction between algal cells and their environment. In light of this, a better understanding of dinoflagellate CWPs composition may help to reveal various physiological activities on the cell wall as well as in the blooming mechanism of dinoflagellates.

Study of CWPs has often relied on the methods used for their isolation from the cell wall of dinoflagellates. However, at present, there is no ideal method for the isolation of CWPs although many studies have been devoted to various membrane proteins. One of the current strategies is to extract CWPs from whole cells using a sequential extraction method [31–33]. However, this approach causes the cells to break during the long chemical extraction, and this results in potential cross-contamination of the CWPs [32]. Specific labeling methods, for example, biotinylation or the use of the radioisotope Na125I, are also developed to recognize and isolate the cell surface proteins (CSPs) from dinoflagellates [26, 30]. However, these methods led to a loss of solubility of the proteins due to the multiple additions of large hydrophobic groups, and, moreover, these methods only address CSPs and not the CWPs.

Global techniques such as proteomics provide effective strategies and tools for profiling and identifying proteins of dinoflagellates [34–38]. In contrast to conventional biochemical approaches that address one or a few specific proteins at a time, proteomic techniques allow simultaneous isolation and identification of hundreds to thousands of proteins in one sample. Fluorescence difference gel electrophoresis (DIGE) technology is a newly developed 2D gel-based approach that employs three fluorescent succinimidyl esters, termed CyDyes, to differentially label proteins prior to electrophoretic separation [39]. Because of the sensitivity and extended linear dynamic range of these dyes, this technique facilitates not only quantification over a comparatively wide dynamic range with high accuracy, but also enables relative quantification with reference to an internal standard, thereby also facilitating the analysis of an adequate set of biological replicates in order to obtain the most significant data on protein regulation. This technique is recently applied for identifying biomarkers, designing novel drug targets, and monitoring therapeutic processes [40–43]. 

In this study, we present a newly developed method for the identification and characterization of CWPs from *A. catenella *DH01, an HAB-causing dinoflagellate species widely spread in the coastal waters of China[44].By comparing sequentially extracted CWPs labeled with Cy3 and protoplast proteins labeled with Cy5, 120 CWPs were confidently identified on the 2D DIGE gel, and the majority gave positive identification of protein orthologues in the protein database by *de novo *sequence analysis and database searching (MS-BLAST). The goal of this study was to establish an efficient and reliable method to identify CWPs from dinoflagellates and to characterize putative proteins in order to provide a foundation for future investigation of the functions and expression of CWPs in *A. catenella* as well as other dinoflagellates.

## 2. Materials and Methods

### 2.1. Organism and Culture Conditions

*A. catenella *DH01 was provided by the Culture Collection Center of Marine Bacteria and Algae of the State Key Laboratory of Marine Environmental Science, Xiamen University, China. A unialgal isolate was routinely maintained in K medium [45] at 20°C under a 10 : 14 h light: dark photoperiod at a light intensity of approximately 100 *μ*mol photons m^−2^ s^−1^ provided using fluorescent lamps. The cells for the experiments were grown in 5,000 mL flasks containing 4,000 mL of K medium, and the culture conditions were the same as above. The K medium did not contain any protein. Approximately 2 × 10^7^ cells of *A. catenella *in their exponential growth phase were collected with centrifugation at 3,000 × g for 30 minutes at 4°C. The cell pellets were rinsed twice with precooled sterilized seawater to avoid any carryover of culture medium and extracellular proteins and were used for the extraction of CWPs and protoplast proteins.

### 2.2. Preparation of Sequentially Extracted CWPs

For CWP extraction, the cell pellets were sequentially extracted with 0.2 M CaCl_2_, 50 mM CDTA in 50 mM sodium acetate (pH 6.5), 2 mM DTT, and 1 M NaCl at 4°C for 30 min each, and finally to 0.2 M borate (pH 7.5) at room temperature for 30 min, with gentle vortexing. The extracts were pooled together and precipitated with three volumes of ice-cold 20% TCA (v/v) in acetone overnight at −20°C and centrifuged at 20,000 g for 30 min at 4°C (Hettich ROTINA 38R Refrigerated Centrifuges, Germany). The supernatant was discarded, and the precipitate was washed twice with ice-cold 90% acetone (v/v) containing 20 mM DTT and then twice with ice-cold 100% acetone. The protein obtained was air-dried to remove residual acetone and subsequently dissolved in 50 *μ*L rehydration buffer (pH 8.5) containing 7 M urea, 2 M thiourea, 4% CHAPS (w/v), and 30 mM Tris and then stored at −80°C for proteomic analysis. This protein sample was termed sequentially extracted CWPs.

### 2.3. Preparation of Protoplast Proteins

**Figure 2:**
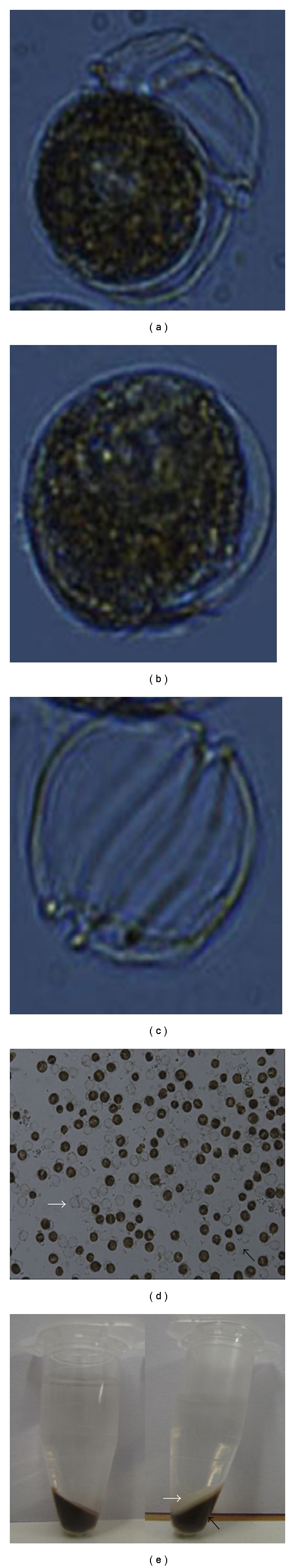
Preparation of protoplasts of *A. catenella *DH01 using the temperature shock method. (a), (b), and (c) are photographs of the intact cell, the protoplast and the cell wall of *A. catenella *DH01 under the light microscope. (d) A mixture of cell walls (white arrow) and protoplasts (black arrow). (e) Concentrated cell walls (white arrow) and protoplasts (black arrow). (The magnitude was 10  ×  20).

1 × 10^7^ cells were resuspended in sterilized sea water and maintained at 4°C for one and a half hours then at 20°C for 10 min. After this treatment, the cell walls became detached from the protoplasts without the cell being broken (Figure 2). The suspension was centrifuged at 4,000 g for 30 min at 4°C. After removing the supernatant, the pellet was separated into two layers, cell walls in the upper layer and protoplasts in the lower layer (Figure 2(e)). The cell wall layer was introduced to a membrane filter of 5 *μ*M diameter pore size (Whatman) with a pipette and gently washed three times with sterilized sea water to avoid contamination by extracellular proteins. The protoplasts were removed on a 10 *μ*M diameter pore size filter (Whatman) and washed three times with sterilized sea water to avoid contamination by the cell walls. 1 mL Trizol reagent was added to the protoplast pellet collected using centrifugation, and it was subjected to sonication on ice. Subsequently, 200 *μ*L of chloroform was added to the cell lysate before shaking it vigorously for 15 s. The mixture was allowed to stand for 5 min at room temperature before being centrifuged at 12000 × g for 15 min at 4°C. The top pale-yellow or colorless layer was removed, and 300 *μ*L of ethanol was added to resuspend the reddish bottom layer and this mixture centrifuged at 2000 × g for 5 min at 4°C. The supernatant was transferred to a new tube and 1.5 mL of isopropanol was added. The mixture was allowed to stand for at least 20 min for precipitation of proteins at room temperature. It was then centrifuged at 14000 × g for 10 min at 4°C, and the pellet obtained was briefly washed with 95% ethanol before being allowed to air dry. 500 *μ*L of rehydration buffer with 7 M urea, 2 M thiourea and 4% W/V CHAPS was added to solubilize the protein pellet before loading onto the first dimension isoelectric focusing (IEF).

### 2.4. Minimal Labeling of Proteins Using Fluorescent Dye

Sequentially extracted CWPs and protoplast proteins were subjected to minimal labeling using the fluorescent dyes Cy3 and Cy5 according to the manufacturer's instructions. Aliquots of 50 *μ*g of each sample were separately labeled. Briefly, stock cyanine dyes (14 nmol/*μ*L) were diluted in freshly prepared DMF to 400 pmol/*μ*L and 8 pmol dye was added per 1 *μ*g of protein in the cell lysate. The sample was vortexed, briefly centrifuged, and left on ice for 30 min in the dark. No primary amines, DTT, or carrier ampholytes were included in the lysis buffer as such components could potentially react with the *N*-hydroxy succinimide ester group of the cyanine dyes. The labeling reaction was quenched by adding 1 *μ*L of 10 mML-lysine per 400 pmol of dye. The sequentially extracted CWPs and protoplast proteins were labeled with Cy3and Cy5, respectively. Thereafter, the Cy3- and Cy5-labeled samples were mixed at a ratio of 1 : 1 (equating to 100 *μ*g of total protein) and prepared for IEF.

### 2.5. Two-Dimensional Gel Electrophoresis

For 2D DIGE, the labeling protein samples were mixed with a rehydration buffer (7 M urea, 2 M thiourea, 4% w/v CHAPS, 1% DTT, and 0.5% v/v IPG) before loading onto IPG strips with a linear pH gradient from 4–7 (Immobiline Drystrip, GE Healthcare Life Science, Piscataway, US). The sample was subjected to IEF using an IPGphor III system with 24 cm IPG strips and the following protocol: 6 h at 40 V (active rehydration), 6 h at 100 V, 0.5 h at 500 V, 1 h at 1000 V, 1 h at 2000 V, 1.5 h at 10000 V, and 6 h at 10000 V for 60000 Vh. The minimal Vh applied was 60000 units. Subsequently, the immobilized pH gradient strips were equilibrated for 15 min in reducing buffer containing 6 M urea, 2% SDS, 50 mM Tris-Cl (pH 8.8), 30% glycerol, and 1% DTT, followed by equilibration for 15 min in alkylation buffer containing 6 M urea, 2% SDS, 50 mM Tris-Cl (pH 8.8), 30% glycerol, and 2.5% iodoacetamide. Second-dimension SDS-PAGE gels (12.5%) were run on a GE Ettan DALT six at 0.5 w/gel for 1 h and then at 17 w/gel for 6 h.

### 2.6. Gel Scanning, Digitizing, and Data Analysis

The resultant analytical gels were scanned using a Typhoon 9400 scanner (Amersham 4 Biosciences/GE Healthcare). The specific excitation and emission wavelengths for each of the fluorescent dyes were recommended by the manufacturer. Gel images were scanned at a resolution of 100 *μ*m and preprocessed using ImageQuant software (version 5.2, Amersham Biosciences/GE Healthcare). Cropped gel images were analyzed using DeCyder 2D software (version 6.5, Amersham Biosciences/GE Healthcare). The differential in-gel analysis (DIA) algorithm detected overlapping spots on a combined image derived from merging individual images from the two samples tagged by Cy3 and Cy5. Protein spots which were identified as CWPs between the sequentially extracted CWPs and protoplast proteins were marked for spot excision and subsequent protein identification using MALDI TOF-TOF.

### 2.7. In-Gel Digestion

120 CWPs identified using 2D DIGE were manually excised from the prepared silver stained 2-DE gels (Figure 4), and the silver-stained gel pieces were rinsed once with MilliQ water and destained in 100 mM sodium thiosulfate and 30 mM potassium ferricyanide until the gel pieces became white. They were then rinsed three times in Milli-Q water, shrunk with 100% acetonitrile for 15 min, and air-dried at room temperature for 30 min. All gel pieces were incubated with 12.5 ng/*μ*L sequencing grade trypsin (Roche Molecular Biochemicals) in 20 mM NH_4_HCO_3_ overnight at 37°C. After digestion, 1 *μ*L of supernatant was pipetted and spotted on the target plate then air-dried at room temperature. 1 *μ*L of matrix (4-hydroxy–cyanocinnamic acid in 30% CAN, 0.1% TFA) was laid over the samples on the target plate until they dried completely.

### 2.8. Mass Spectrometric Analysis

MALDI-TOF mass spectrometry and tandem TOF/TOF mass spectrometry were carried out with a 4800 Plus MALDI TOF-TOF Analyzer (Applied Biosystems, Foster City, USA) equipped with a neodymium: yttrium-aluminum-garnet laser. The laser wavelength and the repetition rate were 355 nm and 200 Hz. The MS spectra were processed using the Peak Explorer (Applied Biosystems) software allowing nonredundant and fully automated selection of precursors for tandem mass spectrometry (MS/MS) acquisition. At least 2000 laser shots were typically accumulated in the MS mode, whereas in the MS/MS mode spectra from up to 5000 laser shots were acquired and averaged. The peak detection criteria used were a minimum S/N of 10, a local noise window width mass/charge (*m*/*z*) of 250 and a minimum full-width half-maximum (bins) of 2.9. The mass spectra were internally calibrated using porcine trypsin autolytic products (*m/z *842.51, 1045.564, and 2211.104 Da) resulting in mass errors of less than 30 ppm. A maximum of the five strongest precursor ions per protein spot were chosen for MS/MS analysis. The following monoisotopic precursor selection criteria were used for MS/MS: minimum S/N filter of 50, excluding the most commonly observed peptide peaks for trypsin and keratin, and excluding the precursors within 200 resolution. In the TOF1 stage, all ions were accelerated to 1 kV under conditions promoting metastable fragmentation. The peak detection criteria used were an S/N of 10 and a local noise window width of 250 (*m*/*z*).

### 2.9. Database Search

A combined MS and MS/MS search was first performed against the NCBI nonredundant database with no taxonomic restriction using an in-house MASCOT server (Version 2.2). The raw MS and MS/MS spectra were processed using GPS Explorer software (Version 3.5, Applied Biosystems). For protein spots with a scores confidence interval below 95%, their MS/MS spectra were used for automated *de novo *sequencing using the Applied Biosystems DeNovo Explorer software [46]. Briefly, each MS/MS spectrum produced ten peptide sequence candidates, and each peptide sequence had a score associated with it that indicated how much of the total ion abundance in the MS/MS spectrum was accounted for by the typical fragment ions that could be calculated for the particular sequence. The closer to 100 was the score, the greater the likelihood that all or most of the sequence generated by DeNovo Explorer was correct. 

*De novo *generated peptide sequences were performed for similarity searches using the MS-BLAST algorithms [46]. The MS-BLAST searches were conducted at the Heidelberg server (http://dove.embl-heidelberg.de/Blast2/msblast.html) against the NCBI nonredundant database using standard settings with no taxonomic restriction. All sequences obtained from an MS/MS spectrum were spaced with the minus symbol (−) and were merged into a single string, and submitted to an MS BLAST search as reported above. The MS-BLAST search results were considered significant if the resulting scores were higher than the threshold score indicated in the MS-BLAST scoring scheme.

## 3. Results

### 3.1. Identification of CWPs Using 2D DIGE

**Figure 3:**
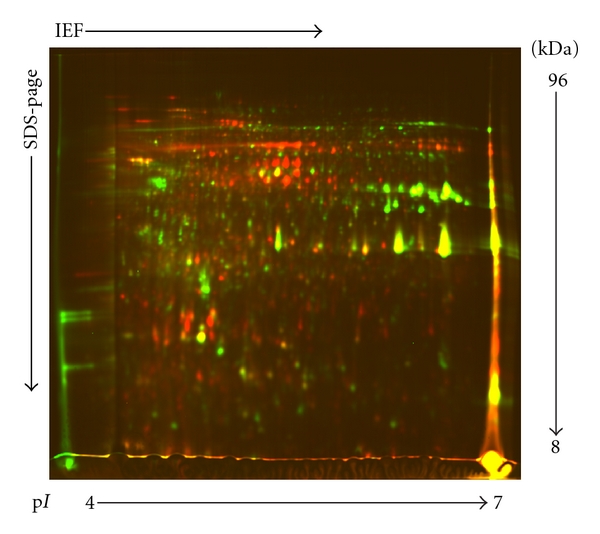
2D DIGE analysis of sequentially extracted CWPs and protoplast proteins labeled using the fluorescent dyes Cy3 (green) and Cy5 (red), respectively. This representative 2D DIGE image for protein expression maps used a 12.5% homogenous SDS-PAGE gel in the pH range 4 to 7.

In this study, two protein fractions were obtained from *A. catenella*: one fraction was sequentially extracted CWPs prepared using a sequential chemical extraction method, a traditional plant CWP preparation method; the other was protoplast proteins prepared using a temperature shock method. The former was labeled with Cy3 and the latter with Cy5, and then, they were pooled together to run 2D DIGE. An overview of the fluorescent DIGE images of the sequentially extracted CWPs and protoplast proteins of *A. catenella*, and the overlaying of these two images are shown in Figure 3. The differentially expressed proteins were evaluated using DeCyder 2D software. This software identifies protein spots and compares the spot intensities for up to three samples run simultaneously on a single 2-DE gel. Figure 3 shows qualitative comparisons of sequentially extracted CWPs and protoplast proteins run on a single gel. Overlaying the images allowed direct comparison of the two. Green spots were sequentially extracted CWPs; red spots were protoplast proteins; yellow spots were the same proteins presented in both sequentially extracted CWPs and protoplast proteins. Using DIA software analysis, 120 candidate protein spots were identified as CWPs (green spots) in the CyDye staining gel, and the majority of these CWPs was separated in the apparent molecular mass range of 14–50 kDa, and they had p*I* ranges of 4.0–7.0.

### 3.2. Categorization of the *A. Catenella* CWPs

**Figure 4:**
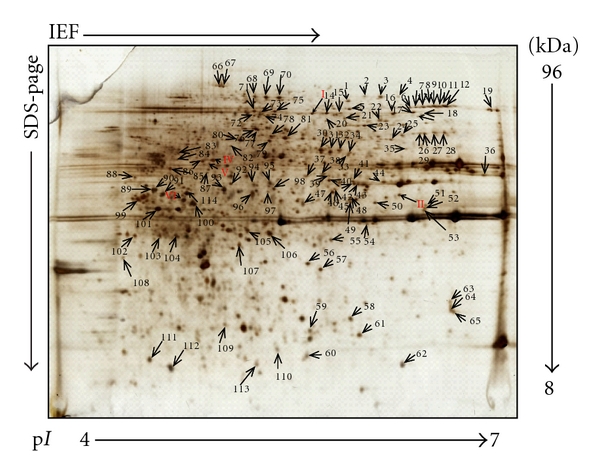
Representative 2-DE gel of CWPs from an *A. catenella *DH01 sample stained with silver. The proteins were resolved in 4–7 linear pH gradient (Immobiline DryStrips; 240 × 3 × 0.5) and 12.5% SDS-PAGE (2400 × 2000 × 1 mm). 120 CWPs were separated and identified (indicated by arrows) from *A. catenella *DH01.

To further characterize the samples, 120 confidently identified CWPs of *A. catenella *DH01 were excised from the silver-staining gels (Figure 4) and trypsinized, before subjection to MALDI-TOF-TOF MS analysis. By searching against the NCBI nonredundant database using the MASCOT algorithm, no CWPs could be identified which were able to meet statistical significance. This is not surprising, however, since no dinoflagellate genome has been established at present. Furthermore, *de novo *sequencing and MS-BLAST similarity searches were used for protein identification. A total of 42 proteins were identified, most of which were associated with cell wall modifying enzymes, cell wall structure, transport/binding, signaling, and defense (Table 1). Among them, 15 proteins were putative cell wall-modifying enzymes, including four hydrolases, two dehydratases, two dehydrogenases, four oxidoreductases, two acyltransferases, and one protease. These proteins are involved in various physiological processes on the cell wall during cell growth and development. Three putative proteins, D-alanyl-D-alanine ligase A, UDP-glucose 4-epimerase and penicillin-binding protein (PBP) were possibly involved in cell wall construction. Transport/binding proteins, and lipoprotein represented another major group of proteins present in the cell wall. Of these, three belonged to ATP-binding cassette (ABC) family, three were other types of transport/binding proteins, and the other was lipoprotein. These proteins were involved in transporting various substrates across the cell wall membranes. The signaling proteins were another important component in CWPs of *A. catenella*, four of them were receptors, one was a binding protein, and two were other signal proteins. Five proteins related to cell defense were identified, they were polymorphic membrane protein B/C family (PMP), dihydropteroate synthase (DHPS), Vpu protein, FmtA-like protein, and SPAC328.04 protein.

In addition to the above proteins, several other proteins such as At2g46420/F11C10.11, CG2962, hypothetical membrane protein, and PB407L were also characterized amongst the CWPs of *A. catenella*, which reflects the roles of CWPs in cell surface physiology and in interactions between cell and environment.

## 4. Discussion

### 4.1. Isolation and Identification of CWPs

In this study, we prepared CWPs using a sequential extraction method. The cells were extracted first with CaCl_2_, then sequentially with CDTA, DTT, borate, and NaCl, which can efficiently extract weak bound, strongly ionically bound, and pectin-bound proteins as well as glycoproteins. This method was successfully used to extract CWPs from suspension-cultured cells of plant species and did not cause contamination of the proteins [31]. However, we found that the extracts became red during the extraction process and a few broken cells were also observed under the microscope (data not shown), indicating that cytosolic proteins might have been released during extraction and so contaminated the CWPs. Continuous extraction with chemical regents might also increase the permeability of the cell wall (theca) membrane and protoplast membrane which would have led to the leakage of intracellular proteins and subsequently contamination of the CWPs. A study on cell wall proteomics of a green alga, *Haematococcus pluvialis*, demonstrates that the sequential extraction method results in contamination of CWPs with intracellular proteins [32]. Several intracellular proteins, RuBisco small subunit orthologue, and ATP synthase-chain orthologue were found in the SDS-PAGE of the CWPs, although the contamination was relatively minor. Thus, the sequential extraction method is not a reliable approach for the extraction of CWPs for a cell wall proteomic study of dinoflagellates, and so, our study used the 2D DIGE method to identify CWPs by combining the sequential extraction method with the protoplast preparation method. The sequentially extracted CWPs were labeled by Cy3, while the protoplast proteins were labeled by Cy5. By comparing the differential expressed proteins to exclude overlayed proteins run on a single gel, the contamination of the intracellular proteins resulting from broken cells was excluded, and the CWPs were confidently identified. This approach provided a reliable and efficient tool to prepare and identify the CWPs of dinoflagellates.

### 4.2. Functions of CWPs in *A. Catenella*

In this study, 42 proteins associated with cell wall-modifying enzymes, cell wall structure, transport/binding, signaling, and defense were tentatively identified from *A. catenella* using *de novo *sequencing and MS-BLAST similarity searches. These proteins reflected their roles in cell wall physiology.

It is known that several reactions (hydrolysis, transglycosylation, transacylation, and redox reactions) are catalyzed by cell wall-modifying enzymes [47, 48]. In our study, 15 putative cell wall-modifying enzymes were identified from the *A. catenella *cell wall, including hydrolases, dehydratases, dehydrogenases, oxidoreductases, acyltransferases, and protease. Hydrolases are classified as EC 3 in the EC number classification of enzymes and catalyze the hydrolysis of various chemical bonds, for example, carbon-nitrogen, ester, and peptide. Various hydrolases are reported in bacteria and higher plant cell walls and play important roles in fruit ripening and tissue softening of plants as well as bacterial germination, vegetative growth, and sporulation [49, 50]. However, little information is available concerning dinoflagellates. In our study, four hydrolases, the carbon-nitrogen family, competence protein comA, BH3453 protein, and probable transmembrane protein, were identified from *A. catenella *cell walls. Two of them are involved in breaking carbon-nitrogen bonds and appear to be involved in the reduction of organic nitrogen compounds and ammonia production. Aside from these hydrolase proteins, a protease, methionine aminopeptidase (MAP), was identified from the cell wall. MAP is responsible for the removal of the amino-terminal (initiator) methionine from nascent eukaryotic cytosolic and cytoplasmic prokaryotic proteins if the penultimate amino acid is small and uncharged. The occurrence of protease in cell walls is reported in bacteria, green algae, and higher plants. In a green alga, *H. pluvialis*, six putative proteases are identified and are postulated to be involved in processing and/or turnover of CWPs during cell growth and development [32].

Two dehydratases, mannonate dehydratase and enolase, were identified from the *A. catenella *cell wall. Mannonate dehydratase belongs to the family of lyases, specifically the hydrolases, which cleave carbon-oxygen bonds and participate in pentose and glucuronate interconversions. Enolase, also known as phosphopyruvate dehydratase, is a metalloenzyme responsible for the catalysis of 2-phosphoglycerate to phosphoenolpyruvate, the ninth and penultimate step of glycolysis. The two enzymes may exert a role in energy provision for cell wall formation.

A number of oxidoreductases were identified amongst the CWPs of *A. catenella*, including Gll1094 protein, 5, 10-methylenetetrahydrofolate reductase, tyrosinase, and gamma-glutamyl phosphate reductase. Aside from these proteins, two dehydrogenases (ethanol dehydrogenase and acyl-CoA dehydrogenase) were also detected in *A. catenella*. Acyl-CoA dehydrogenase catalyzes the initial step in each cycle of fatty acid *β*-oxidation and results in the introduction of a trans double bond between C2 and C3 of the acyl-CoA thioester substrate. Recently, several oxidoreductases, such as peroxidase, peptide Met (O) reductase 3, cytokinin oxidase, thioredoxin H-type 5, and UDP-*N*acetylmuramate-dehydrogenase, are identified in *H. pluvialis *cell wall extract [32]. It is suggested that oxidoreductases might cause reduction of cell wall extensibility by forming bridges across phenolic residues and adjacent CWPs or polysaccharides [51]. 

Two acyltransferases, phosphotransacetylase and 8-amino-7-oxononanoate synthase, were found in *A. catenella*. These two enzymes belong to the family of transferases, specifically the acyltransferase transferring groups rather than the aminoacyl groups. The former participates in taurine and hypotaurine metabolism, pyruvate metabolism, and propanoate metabolism, while the latter participates in biotin metabolism. Both of them might play important roles in the formation and stability maintenance of the cell wall. 

Three putative proteins identified in this study were possibly involved in cell wall construction. PBP is the primary enzyme involved in cell wall biosynthesis including muramoylpentapeptide carboxypeptidase, peptide syntheses, transpeptidases, and hexosyltransferases. In bacteria, PBP is involved in the final stage of the synthesis of peptidoglycan, the major component of bacterial cell walls. Occurrence of the three proteins suggested that the cell wall of dinoflagellates may contain components similar to bacterial peptidoglycan, which can form a strong and rigid lattice-like structure. Recently, three proteins associated with cell wall construction, S-layer protein, cellulose synthase, and 1UDP-N-acetylmuramoyl-alanine-D-glutamateligas, were identified from a green alga *H. pluvialis *[32]. Moreover, cell division inhibitor MinD, a peripheral protein, was identified in our study. MinD is a ubiquitous ATPase that plays a crucial role in the selection of the division site in eubacteria, chloroplasts, and probably Archaea and cooperates with MinC to form a division inhibitor at the cell division site that is topologically regulated by MinE. Recently, MinD has been found in four green algae, and the overexpression of MinD results in the MinCD complex binding all cell division sites and inhibiting cell division and leads to a long and nonseptate filamentous cell [52]. Moreover, MinD affects the diameters of cells. Since in dinoflagellates with a theca (amphiesma) little is known about the cell wall biogenesis and dynamics, identification of MinD suggested that this protein might act as a mediator to regulate cell wall growth and cell size when exposed to environmental stresses. 

Seven putative transport/binding proteins and lipoprotein represented another major group of proteins present in the cell wall of *A. catenella*. ABC family proteins are transmembrane proteins that utilize the energy of ATP hydrolysis to carry out various biological processes including translocation of various substrates across membranes, and nontransport-related processes such as translation of RNA and DNA repair. They transport a wide variety of substrates across extra- and intracellular membranes, including metabolic products, lipids and sterols, and drugs. Recently, six ATP-binding cassette transporters are identified in the cell wall of *H. pluvialis *[32]. In *Synechocystis*, ABC-type transporters represent the most abundant transporters in the periplasmic space that are involved in the uptake of inorganic nutrients [33]. These studies indicate that ATP-binding cassette transporters might play important roles in the nutrient transport of dinoflagellates. Aside from these proteins, outer membrane lipoprotein OMP 16, similar to uniprot P40548 and calcium channel alpha-1 subunit homolog, were also identified in the CWPs of *A. catenella*, and these proteins played important roles in protein binding, lipid anchor, and calcium binding of the cell walls. Interestingly, luciferin-binding protein, a protein involved in the bioluminescence reaction, was identified from the CWPs of *A. catenella*. It is interesting to note that most of the proteins described above were previously found to be associated with the plasma membrane. This suggests that potential direct physical connections may occur between the plasma membrane and the cell wall and/or interactions at the plasma-cell wall interface [53]. 

The signaling proteins are another important component in plant cell walls, which regulate various biological processes occurring in the cell wall, such as signal transduction, cell shape and size regulation, stress response, and defense. Melanocortin 4 receptor and G-protein receptor are two transmembrane receptors that sense molecules outside the cell and activate inside signal transduction pathways and, ultimately, cellular responses. G protein-coupled receptors are found only in eukaryotes. Translocon-associated protein beta and tyrosine kinase negative regulator Cb1 are a signal sequence receptor and a cell surface receptor linked to signal transduction, respectively. Guanine nucleotide-binding proteins are glycoproteins anchored on the cytoplasmic cell membrane. They are mediators for many cellular processes, including signal transduction, protein transport, growth regulation, and polypeptide chain elongation. They are also known as GTP-binding proteins and GTPases. Almost all members of this super family of proteins act as a molecular switch, which is on when GTP is bound and off when GDP is bound. CG34393 was involved in regulation of small GTPase-mediated signal transduction. Our study also found one light signal transduction protein, Cg1 protein, which is a light induced protein and regarded as a possible member of a light signal transduction chain in parsley [54], indicating that Cg1 protein might function as a light-driven proton pump and take advantage of light energy directly as proteorhodopsin in *A. catenella*.

Proteins related to cell defense were also identified in cell wall of *A. catenella*. PMP is a bacterial outer membrane protein which might play important roles in the growth and development of *Chlamydia pneumoniae *[55]. DHPS, which has been found in bacteria, is a key enzyme in producing dihydropteroate. In the lower eukaryote *Pneumocystis carinii*, DHPS is the C-terminal domain of a multifunctional folate synthesis enzyme [56]. Finding DHPS in *A. catenella *suggested that this protein might have originated from the symbiotic bacteria which are hosted on the surface of *A. catenella *cells. Vpu protein and FmtA-like protein are two important proteins which play important roles in resisting bacteria and viruses.

### 4.3. Protein Identification Using De Novo Analysis and Database Searching

*De novo *analysis coupled with database searching is regarded as a powerful proteomic technique for protein identification, particularly for species with an unknown or incomplete genome [57–59]. Comparative genomic and proteomic studies have demonstrated that the amino acid sequence of proteins is significantly conserved across species boundaries. The conserved nature of many biosynthetic, metabolic, and regulatory pathways in different organisms was the basis for earlier studies of cross-species protein identification for species whose genome sequence were unknown or incomplete. Molecular information on cell wall biogenesis and dynamics of dinoflagellates is totally lacking due to the lack of a dinoflagellate genome at the present. For example, not a single CWP has been identified from dinoflagellates. In this study, approximately two thirds of the tested protein spots failed to be characterized in the protein databases, which might be caused by the low sequence homology matching for unambiguous protein identification across species boundaries. Studies have shown that amino acid residue substitutions occur in many positions of a specific protein across species boundaries resulting from evolutionary divergence as well as numerous posttranslational modifications, for example, phosphorylation, glycosylation, and acetylation, which might reduce or diminish the probability or efficiency of cross-species identification. In addition, low abundance and limited number of CWP sequences present in the available databases might further contribute to the limitations of the technique. 

In summary, our study provided a newly developed method for identifying and characterizing CWPs from *A. catenella*. By combining the sequential extraction method for CWPs and the protoplast preparation method, the CWPs were separated from cytosolic proteins using the 2D DIGE method. This method has the potential to become a reliable complement to other methods currently used in studies of dinoflagellate CWPs. As a preliminary study, 120 CWPs were recognized, and 42 were characterized, such as cell wall-modifying enzymes, cell wall structural proteins, transport/binding proteins, signaling, and defense proteins. More insights can be expected; for example, the rapid analysis of many CWPs as well as the characterization of the proteomic changes occurring at the cell wall in response to environmental stresses are expected to facilitate the identification of new surface-exposed targets, and this can certainly improve our understanding of the relationship between cells and environmental variations.

## Figures and Tables

**Table 1:** Functional categorization of CWPs from *A. catenella* DH01.

Spot no.	Accession no.	Identification of MS-blast	MS-blast score (HSPs)
Cell wall modifying enzymes			

III	AACY01006738	Quinoprotein ethanol dehydrogenase	114 (2)
V	Q7S9I3	Acyl-CoA dehydrogenase	107 (2)
5	Q9F1X6	Phosphotransacetylase	100 (2)
8	Q8XTK4	Probable transmembrane protein	135 (3)
24	Q5WKD5	Mannonate dehydratase	104 (2)
27	Q635P5	Hydrolase, carbon-nitrogen family	59 (1)
37	Q7NLM8	Gll1094 protein	65 (1)
43	Q88D51	5,10-methylenetetrahydrofolate reductase	80 (1)
46	D00131	Tyrosinase	73 (1)
66	CP000025	8-amino-7-oxononanoate synthase	64 (1)
69	P51973	Competence protein comA	65 (1)
80	Q63QT9	Gamma-glutamyl phosphate reductase	107 (2)
81	Q5UU97	Enolase	141 (3)
98	Q9K7B3	BH3453 protein	102 (2)
102	Q72CF9	Methionine aminopeptidase	64 (1)

Transport/binding proteins and lipoproteins			

I	Q39909	Luciferin-binding protein	110 (2)
17	O73697	Calcium channel alpha-1 subunit homolog	75 (1)
36	Q6FPN9	Similar to uniprot∣P40548 Saccharomyces cerevisiae YIL016w SNL1	76 (1)
85	Q926C3	Outer membrane lipoprotein omp16 homolog	97 (2)
97	CP000009	Carbamoyl-phosphate synthase large chain	70 (1)
100	Q833S0	ABC transporter, ATP-binding protein	97 (2)
112	Q6IV89	F1Fo-ATPase synthase f subunit	59 (1)

Signaling proteins			

II	Q01369	Guanine nucleotide-binding protein beta subunit-like protein	152 (3)
18	Q7T0K6	Melanocortin 4 receptor	64 (1)
25	CAAJ01000020	Cg1 protein, putative	70 (1)
57	Q6WQQ4	Translocon-associated protein beta	67 (1)
72	AE006464	possible G-protein receptor	99 (2)
88	Q9VQM8	CG34393	68 (1)
103	Q98TY6	Tyrosine kinase negative regulator Cbl	137 (3)

Cell wall structure-related proteins			

VI	Q8H6H9	Cell division inhibitor MinD	66 (1)
1	Q6N415	Putative D-alanyl-D-alanine ligase A	105 (2)
99	Q82D96	Putative UDP-glucose 4-epimerase	64 (1)
111	*C2Q9T5*	Penicillin-binding protein	66 (1)

Defense			

IV	AE002181	polymorphic membrane protein B/C family	67 (1)
31	Q9WXP7	Dihydropteroate synthase	101 (2)
63	Q9Q6Y7	Vpu protein	65 (1)
74	Q8TR39	FmtA-like protein	70 (1)
109	Q9P3U2	SPAC328.04 protein	60 (1)

Uncharacterized proteins			

32	Q9SKD8	At2g46420/F11C10.11	106 (2)
49	Q9W2X5	CG2962	60 (1)
15	Q89W76	hypothetical membrane protein	73 (1)
78	Q65173	PB407L	104 (2)
